# Supplementary material for: Unveiling the Bioactive Potential of the Invasive Jellyfish Phyllorhiza punctata Through Integrative Transcriptomic and Proteomic Analyses
Source: Biomolecules. 2025 Aug 4;15(8):1121. doi: 10.3390/biom15081121 (PMC12383608; doi:10.3390/biom15081121)
Supplement: Supplementary file 1 [file biomolecules-15-01121-s001.zip › Suplementary Material/FileS3/PEPFOLD4/XP_065052055_7837/clusters.html]

 PEP-FOLD clusters


Download archive of all models  here   

|  |  |  |
| --- | --- | --- |
| # | file | sOPEP |
| # cluster 1 - Size: 67 |  |  |
|  | XP\_065052055\_7837-model1 | -39.8065 |
|  | XP\_065052055\_7837-model1.1 | -39.7838 |
|  | XP\_065052055\_7837-model1.2 | -39.7528 |
|  | XP\_065052055\_7837-model1.3 | -39.734 |
|  | XP\_065052055\_7837-model1.4 | -39.5905 |
|  | XP\_065052055\_7837-model1.5 | -39.5783 |
|  | XP\_065052055\_7837-model1.6 | -39.4417 |
|  | XP\_065052055\_7837-model1.7 | -39.4107 |
|  | XP\_065052055\_7837-model1.8 | -39.2246 |
|  | XP\_065052055\_7837-model1.9 | -39.2092 |
|  | XP\_065052055\_7837-model1.10 | -39.1823 |
|  | XP\_065052055\_7837-model1.11 | -39.1229 |
|  | XP\_065052055\_7837-model1.12 | -39.0868 |
|  | XP\_065052055\_7837-model1.13 | -38.9784 |
|  | XP\_065052055\_7837-model1.14 | -38.9656 |
|  | XP\_065052055\_7837-model1.15 | -38.9518 |
|  | XP\_065052055\_7837-model1.16 | -38.9435 |
|  | XP\_065052055\_7837-model1.17 | -38.9227 |
|  | XP\_065052055\_7837-model1.18 | -38.9198 |
|  | XP\_065052055\_7837-model1.19 | -38.8885 |
|  | XP\_065052055\_7837-model1.20 | -38.8876 |
|  | XP\_065052055\_7837-model1.21 | -38.8636 |
|  | XP\_065052055\_7837-model1.22 | -38.8478 |
|  | XP\_065052055\_7837-model1.23 | -38.8211 |
|  | XP\_065052055\_7837-model1.24 | -38.817 |
|  | XP\_065052055\_7837-model1.25 | -38.799 |
|  | XP\_065052055\_7837-model1.26 | -38.7593 |
|  | XP\_065052055\_7837-model1.27 | -38.738 |
|  | XP\_065052055\_7837-model1.28 | -38.7357 |
|  | XP\_065052055\_7837-model1.29 | -38.7243 |
|  | XP\_065052055\_7837-model1.30 | -38.6974 |
|  | XP\_065052055\_7837-model1.31 | -38.6597 |
|  | XP\_065052055\_7837-model1.32 | -38.6444 |
|  | XP\_065052055\_7837-model1.33 | -38.6313 |
|  | XP\_065052055\_7837-model1.34 | -38.6147 |
|  | XP\_065052055\_7837-model1.35 | -38.586 |
|  | XP\_065052055\_7837-model1.36 | -38.5691 |
|  | XP\_065052055\_7837-model1.37 | -38.4146 |
|  | XP\_065052055\_7837-model1.38 | -38.4076 |
|  | XP\_065052055\_7837-model1.39 | -38.3677 |
|  | XP\_065052055\_7837-model1.40 | -38.3632 |
|  | XP\_065052055\_7837-model1.41 | -38.3406 |
|  | XP\_065052055\_7837-model1.42 | -38.3361 |
|  | XP\_065052055\_7837-model1.43 | -38.3219 |
|  | XP\_065052055\_7837-model1.44 | -38.3159 |
|  | XP\_065052055\_7837-model1.45 | -38.3046 |
|  | XP\_065052055\_7837-model1.46 | -38.2927 |
|  | XP\_065052055\_7837-model1.47 | -38.2796 |
|  | XP\_065052055\_7837-model1.48 | -38.2769 |
|  | XP\_065052055\_7837-model1.49 | -38.2585 |
|  | XP\_065052055\_7837-model1.50 | -38.256 |
|  | XP\_065052055\_7837-model1.51 | -38.2068 |
|  | XP\_065052055\_7837-model1.52 | -38.1773 |
|  | XP\_065052055\_7837-model1.53 | -38.173 |
|  | XP\_065052055\_7837-model1.54 | -38.1516 |
|  | XP\_065052055\_7837-model1.55 | -38.1352 |
|  | XP\_065052055\_7837-model1.56 | -38.1311 |
|  | XP\_065052055\_7837-model1.57 | -38.1222 |
|  | XP\_065052055\_7837-model1.58 | -38.0094 |
|  | XP\_065052055\_7837-model1.59 | -37.9307 |
|  | XP\_065052055\_7837-model1.60 | -37.9137 |
|  | XP\_065052055\_7837-model1.61 | -37.674 |
|  | XP\_065052055\_7837-model1.62 | -37.4982 |
|  | XP\_065052055\_7837-model1.63 | -37.4589 |
|  | XP\_065052055\_7837-model1.64 | -37.1063 |
|  | XP\_065052055\_7837-model1.65 | -37.0515 |
|  | XP\_065052055\_7837-model1.66 | -37.0143 |
| # cluster 2 - Size: 30 |  |  |
|  | XP\_065052055\_7837-model2 | -38.0969 |
|  | XP\_065052055\_7837-model2.1 | -37.8646 |
|  | XP\_065052055\_7837-model2.2 | -37.6913 |
|  | XP\_065052055\_7837-model2.3 | -37.6672 |
|  | XP\_065052055\_7837-model2.4 | -37.5933 |
|  | XP\_065052055\_7837-model2.5 | -37.5698 |
|  | XP\_065052055\_7837-model2.6 | -37.1788 |
|  | XP\_065052055\_7837-model2.7 | -37.1563 |
|  | XP\_065052055\_7837-model2.8 | -37.1525 |
|  | XP\_065052055\_7837-model2.9 | -37.128 |
|  | XP\_065052055\_7837-model2.10 | -37.1219 |
|  | XP\_065052055\_7837-model2.11 | -37.0763 |
|  | XP\_065052055\_7837-model2.12 | -36.9738 |
|  | XP\_065052055\_7837-model2.13 | -36.7955 |
|  | XP\_065052055\_7837-model2.14 | -36.7856 |
|  | XP\_065052055\_7837-model2.15 | -36.749 |
|  | XP\_065052055\_7837-model2.16 | -36.7238 |
|  | XP\_065052055\_7837-model2.17 | -36.6839 |
|  | XP\_065052055\_7837-model2.18 | -36.6705 |
|  | XP\_065052055\_7837-model2.19 | -36.6642 |
|  | XP\_065052055\_7837-model2.20 | -36.5954 |
|  | XP\_065052055\_7837-model2.21 | -36.5852 |
|  | XP\_065052055\_7837-model2.22 | -36.5742 |
|  | XP\_065052055\_7837-model2.23 | -36.4459 |
|  | XP\_065052055\_7837-model2.24 | -36.4318 |
|  | XP\_065052055\_7837-model2.25 | -36.3871 |
|  | XP\_065052055\_7837-model2.26 | -36.3492 |
|  | XP\_065052055\_7837-model2.27 | -36.3239 |
|  | XP\_065052055\_7837-model2.28 | -36.2348 |
|  | XP\_065052055\_7837-model2.29 | -36.1027 |
| # cluster 3 - Size: 2 |  |  |
|  | XP\_065052055\_7837-model3 | -37.1079 |
|  | XP\_065052055\_7837-model3.1 | -36.3933 |
| # cluster 4 - Size: 1 |  |  |
|  | XP\_065052055\_7837-model4 | -35.9739 |
|  |  |  |
